# Improving the Activity of Trp-Rich Antimicrobial Peptides by Arg/Lys Substitutions and Changing the Length of Cationic Residues

**DOI:** 10.3390/biom8020019

**Published:** 2018-04-19

**Authors:** Mauricio Arias, Kathlyn B. Piga, M. Eric Hyndman, Hans J. Vogel

**Affiliations:** 1 Biochemistry Research Group, Department of Biological Sciences, University of Calgary, Calgary, AB T2N 1N4, Canada; ariasm@ucalgary.ca (M.A.); kathlyn.piga@ucalgary.ca (K.B.P.); 2 Physics School, Faculty of Science, Universidad Nacional de Colombia, Medellín, Antioquia 050034, Colombia; 3 Department of Surgery, Division of Urology, Southern Alberta Institute of Urology, University of Calgary, Calgary, AB T2V 1P9, Canada; erichyndman@shaw.ca

**Keywords:** antimicrobial peptides, tritrpticin, arginine, lysine, protease degradation, trypsin

## Abstract

Antimicrobial peptides (AMPs) constitute a promising alternative for the development of new antibiotics that could potentially counteract the growing number of antibiotic-resistant bacteria. However, the AMP structure–function relationships remain unclear and detailed studies are still necessary. The positively charged amino acid residues (Arg and Lys) play a crucial role in the activity of most AMPs due to the promotion of electrostatic interactions between the peptides and bacterial membranes. In this work we have analyzed the antimicrobial and structural properties of several Trp-rich AMPs containing exclusively either Arg or Lys as the positively charged residues. Their antimicrobial activity and mechanism of action were investigated, showing that Lys residues give rise to a reduced antimicrobial potency for most peptides, which was correlated, in turn, with a decrease in their ability to permeabilize the cytoplasmic membrane of *Escherichia coli*. Additionally, the presence of Arg and Lys renders the peptides susceptible to degradation by proteases, such as trypsin, limiting their therapeutic use. Therefore, modifications of the side chain length of Arg and Lys were investigated in an attempt to improve the protease resistance of AMPs. This approach resulted in enhanced stability to trypsin digestion, and in several cases, shorter sidechains conserved or even improved the antimicrobial activity. All together, these results suggest that Arg-to-Lys substitutions, coupled with side chain length modifications, can be extremely useful for improving the activity and stability of AMPs.

## 1. Introduction

Antimicrobial peptides (AMPs) are short amino acid sequences (5–50 residues) that are part of the innate immune systems of multicellular organisms, and they are involved in the protection of the host against infections by microorganisms [1,2,3,4]. In most cases this protection is achieved through different approaches, including direct killing, growth inhibition, and activation and/or modulation of the host immune response [5,6,7]. In the case of direct killing and growth inhibition, all AMPs are required to initially interact with the membrane of the microorganism. This interaction can then lead to membrane destabilization/permeabilization and/or peptide internalization allowing subsequent interactions with intracellular targets, such as DNA, RNA, or enzymes [1,2,8]. 

Due to the key role of peptide–membrane interactions in the mechanism of action of AMPs, numerous structure–function studies have focused on these interactions in order to gain knowledge about the role of specific amino acid residues in the activity of the peptides. The positively charged residues (Arg and Lys) have been shown to be extremely important for the activity of the peptides, due to their ability to promote electrostatic attraction with the negatively charged membrane of microorganisms such as bacteria [9,10]. Similarly, bulky hydrophobic residues, including Trp, have been shown to play a key role for most AMPs in the hydrophobic interactions that occur subsequent to the initial electrostatic attraction [11,12]. However, the same residues that are essential for the activity of AMPs also constitute an Achilles’ heel when they are considered for systemic use against infections. The low stability of most peptides in biological fluids, such as serum or the gastrointestinal tract [13,14], is in part due to the susceptibility of AMPs to degradation by proteases. Serine proteases such as trypsin are highly specific for Arg and Lys residues, while chymotrypsin recognizes aromatic residues (Trp, Phe, and Tyr). Other proteases such as elastase and the aspartate protease pepsin are specific for hydrophobic residues such as Val, Ala, Phe, and Leu [15]. Several strategies have been used to reduce the proteolytic susceptibility of AMPs [14,16], including end-terminal and backbone or side chain modifications, peptide cyclization [17], α/β-peptides, and synthesis of all d-peptide isomers [18,19]. Also, modification of the side chain length of positively charged amino acids has been studied previously for specific AMPs, mainly in an attempt to promote proteolysis resistance against trypsin [13,15,20,21], in blood serum [17,18,19] or in whole organ extracts [13]. These studies have shown that changes in the number of methylene groups of the side chain of Arg and Lys can result in increased resistance towards tryptic degradation.

Due to the dual role that Arg and Lys play in the activity and protease susceptibility of AMPs, we have focused in this work on the role of these cationic residues in the activity, mechanism of action, and proteolytic stability of a diverse group of Trp-rich AMPs. The differences in the chemical nature of the side chain groups for Arg (guanidinium) and Lys (amino) confer distinct properties to the peptides, affecting their antimicrobial activity. In most cases the presence of Lys has been associated with a reduction in the antimicrobial activity of the peptides. This behavior can be explained by the higher number of hydrogen bonds that can be formed by the guanidinium group in contrast to the amino group [22,23,24]. However, to the best of our knowledge, potential correlations between the antimicrobial activity and the effects on the mechanism of action have not yet been addressed in detail for a diverse group of AMPs. Our results are in agreement with other literature reports of lower antimicrobial activity due to the presence of Lys as the sole positively charged residues. Remarkably, the reduction of the antimicrobial activity could be correlated with a decrease in the ability of Lys-containing peptides to induce membrane permeabilization in liposomes and in the cytoplasmic membrane of *Escherichia coli* for most AMPs. 

In addition, modifications of the side chain length of the Arg and Lys residues were introduced in order to establish the role of these amino acid analogs, not only in the proteolytic stability against trypsin, but also in the antimicrobial activity and the capacity to permeabilize artificial and bacterial membranes. This part of our study demonstrated that the modification of the side chain length for Arg and Lys residues in tritrpticin drastically increases the stability of the peptides when in the presence of trypsin. Intriguingly, reduction of the side chain length of the Lys residues also led to an increase in the antimicrobial activity, providing an additional tool to improve the activity of AMPs. 

## 2. Results

### 2.1. Peptide Design

A diverse group of Trp-rich peptides was selected for this study in order to encompass a relatively broad group of AMPs (Table 1). Trp-rich peptides are amongst the shortest AMPs, and they have different sequences and structures, allowing us to study the effects of different positively charged residues in a more general context, including diverse biochemical properties and antimicrobial potencies. For comparison, the well-studied helical peptide magainin was also included in this study. Special emphasis was given to Tritrp-derived peptides, as these peptides have been extensively studied and several analogs have already been described, and their structural and functional characteristics are well established [25].

**Figure 1:**
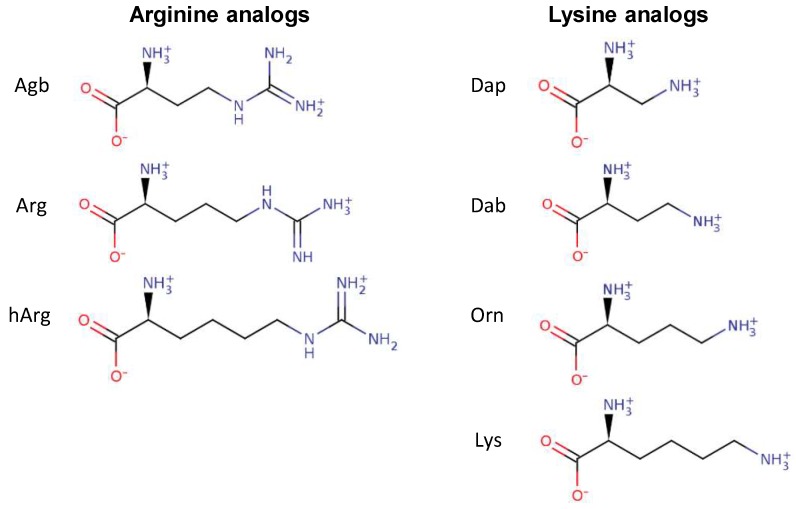
Chemical structures of the amino acids arginine (Arg) and lysine (Lys) as well as their analogs with different side chain lengths. Abbreviations: Agb, (*S*)-2-amino-4-guanidinobutyric acid; hArg, homo-arginine; Dap, 2,3-diaminopropionic acid; Dab, 2,4-diaminobutyric acid; Orn, ornithine.

Several peptide analogs with Arg or Lys residues as the sole source of positive charge were studied in order to assess the effect of the chemical nature of these positively charged residues on the antibacterial activity of several AMPs. The Arg residue is characterized by a guanidinium group at the end of its side chain, while the Lys residues possess an amino group (Figure 1). In addition to changing the nature of the side chain, the length of these side chains was also modified. In the case of Arg, the side chain contains three aliphatic carbons. Two available analogs of Arg that were used in this study included (*S*)-2-amino-4-guanidinobutyric acid (Agb) and homo-arginine (hArg), containing two and four carbons in the side chain, respectively (Figure 1). In the case of Lys, four aliphatic carbons are part of its side chain. Shorter versions of Lys that were studied here included ornithine (Orn) (3-carbons), 2,4-diaminobutyric acid (Dab) (2-carbons), and 2,3-diaminopropionic acid (Dap) (1-carbon) (Figure 1).

### 2.2. Antibacterial Activity

The antibacterial activity of the Tritrp-derived peptides and other AMPs was assessed against *E. coli* ATCC 25922, as a representative of Gram-negative bacteria, incubated in Mueller-Hinton broth media (Table 2). All peptides tested exhibited antibacterial activity in the low µM range—in some cases similar to the activity of the highly toxic peptide melittin (minimal inhibitory concentration (MIC) 2 µM). Arg-to-Lys mutations were correlated with a decrease in activity for almost all peptides. Only the activity of the helical Pro-to-Ala-substituted Tritrp3 peptide (2 µM) was not affected by this substitution, while the peptide PuroA exhibited a slightly higher activity when the positively charged residues were Lys instead of Arg. The lack of sensitivity of the activity of the Tritrp3 peptide to the nature of the positively charged residues prompted a more detailed study of the structure-activity relationship. Tritrp7 and Tritrp8 are analogs of Tritrp3 where only a single Pro residue is mutated by Ala. These mutations are known to alter the 3D structure of the peptides when interacting with sodium dodecyl sulfate (SDS) micelles, specifically reducing the helicity of the original Tritrp3 peptide [25] (Table 1). Mutation of Pro-5 to Ala (Tritrp7) resulted in a peptide that was highly susceptible to the nature of the positively charged residues, with Tritrp7–Arg exhibiting an MIC of 8 µM and Tritrp7–Lys of 32 µM. In contrast, mutation of Pro-9 (Tritrp8) resulted in a peptide that is less affected by the nature of the positively charged residues present, with MICs of 2–4 µM (Table 2). 

The effect induced by changes in the side chain length of Arg and Lys residues was also evaluated for the Tritrp peptides (Table 2). In the case of the Arg analogs, the reduction (Tritrp–Agb) or increase (Tritrp–hArg) of the side chain length did not have a large influence on the antibacterial activity (MIC 2–4 µM) of the peptides. However, the reduction of the side chain length for the Lys residue resulted in a length-dependent increase of the antibacterial activity of the peptides. For the peptide with the shortest sidechain (Tritrp–Dap) the antibacterial activity was 4 µM, while the peptide with the longer (four carbons) Lys residue (Tritrp–Lys) exhibited an MIC of 16 µM for its activity. Intermediate lengths (2–3 carbons) for the side chain of Lys resulted in peptides with antibacterial activity values between 4 and 16 µM.

### 2.3. Escherichia coli Inner Membrane Permeabilization

**Figure 2:**
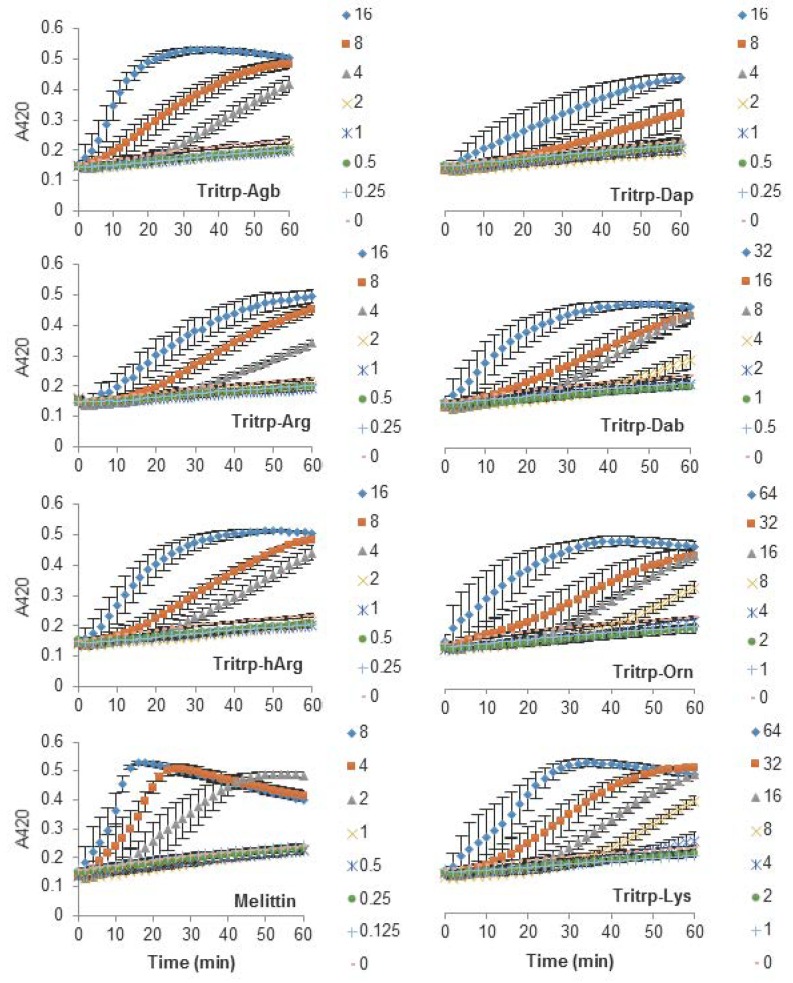
Permeabilization of the inner membrane of *Eschericia coli* ML35p by the action of several Tritrp analogs with different Arg and Lys side chain lengths. Bacterial cells (optical density (OD)_600_ = 0.3) were incubated at 37 °C for 1 h in the presence of peptides at concentrations (µM) corresponding to the values indicated by the legends on the right. Results are average ± standard error of the mean (S.E.M.) (*n* = 4). Melittin was used as a control peptide.

**Figure 3:**
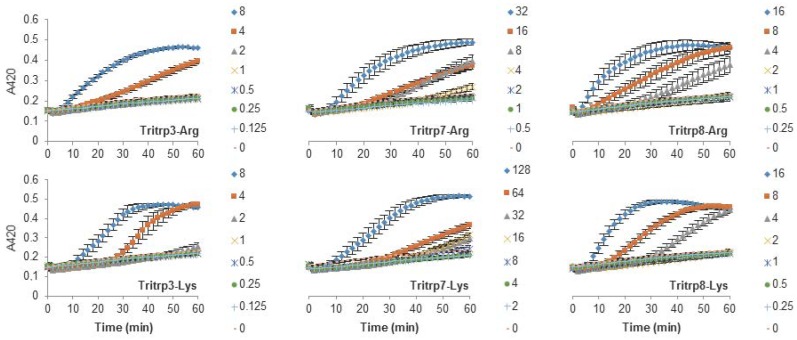
Permeabilization of the inner membrane of *E. coli* ML35p by the action of several Tritrp analogs with Pro-to-Ala and Arg-to-Lys substitutions. Bacterial cells (OD_600_ = 0.3) were incubated at 37 °C for 1 h in the presence of peptides at concentrations (µM) corresponding to the values indicated by the legends on the right. Results are average ± S.E.M. (*n* = 4).

**Figure 4:**
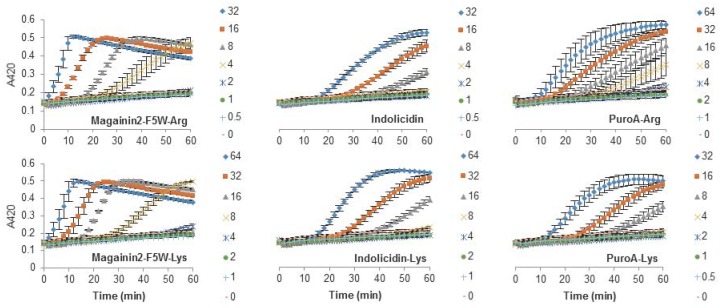
Permeabilization of the inner membrane of *E. coli* ML35p by the action of several Trp-rich AMPs and magainin2-F5W with Arg-to-Lys mutations. Bacterial cells (OD_600_ = 0.3) were incubated at 37 °C for 1 h in the presence of peptides at concentrations (µM) corresponding to the values indicated by the legends on the right. Results are average ± S.E.M. (*n* = 3).

The antibacterial activity of many AMPs has often been related to their ability to induce membrane permeabilization [2,10]. In the case of Gram-negative bacteria, such as *E. coli*, the outer and inner membrane constitute a potential target for the activity of the peptides. In this study, the permeabilization of the inner membrane was assessed in order to establish the role of the disruption of the membrane permeability in the activity of the peptides (Figure 2, Figure 3 and Figure 4). The concentrations used were setup as a twofold dilution from 4×MIC as established for *E. coli* ATCC 25922 (Table 2). Most peptides were capable of inducing inner membrane permeabilization at the MIC (Figure 2, Figure 3 and Figure 4, gray triangle), while peptides such as Tritrp–Dap, Tritrp3–Arg and Tritrp3–Lys only induced permeabilization at 2 × MIC (Figure 2 and Figure 3, orange square). 

The Arg-to-Lys mutations were normally associated with an increase in the peptide concentration required to induce inner membrane permeabilization (Figure 2, Figure 3 and Figure 4). However, peptides such Tritrp3, Tritrp8, and PuroA exhibited similar levels of membrane permeabilization at the same concentrations (Figure 3 and Figure 4). 

The length of the side chain of the Arg and Lys residues had a different effect depending on the nature of the end group in the residue. For the guanidinium group in Arg, changes in the number of carbons in the side chain did not have a substantial effect on the inner membrane permeabilization properties of the peptides (Figure 2, left). In contrast, peptides containing Lys analogs were strongly affected by the number of carbons in the side chain (Figure 2, right). Decreasing the length of the Lys analog residue led to a higher concentration of peptide being required to induce similar levels of permeabilization. Tritrp–Dap (with only one carbon in its side chain) exhibited the lowest levels of membrane permeabilization even at 2×MIC after one hour of incubation (Figure 2, right). In comparison, the peptide with the longest side chain (Tritrp–Lys), with four carbons in its side chain, was able to induce membrane permeabilization at half the MIC. 

### 2.4. Large Unilamellar Vesicle Membrane Permeabilization

**Figure 5:**
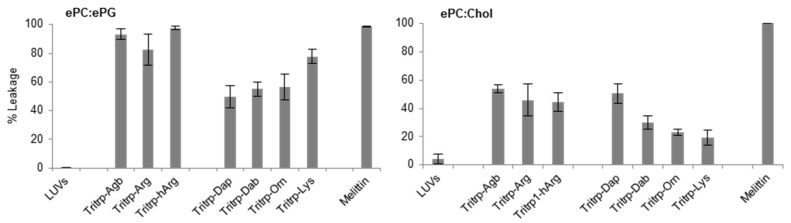
Calcein leakage induced by analogs of the AMP Tritrp and melittin against large unilamellar vesicles (LUVs) composed of egg-derived phosphatidyl-choline (ePC)/egg-derived phosphatidyl-glycerol (ePG) (1:1) and ePC/cholesterol (Chol) (2.5:1). Peptides (0.2 µM) containing different numbers of aliphatic carbons in the Arg or Lys side chain where incubated in the presence of LUVs (2 µM) for 15 min at 25 °C. Results are average ± standard deviation (S.D.) (*n* = 3).

The permeabilization of large unilamellar vesicle (LUV) membranes induced by all the Tritrp-derived peptides was assessed by measuring the calcein leakage [32]. In order to emulate the different surface charges that are characteristic of bacterial and eukaryotic cell membranes, two different LUV systems were prepared. Egg-derived phosphatidyl-glycerol (ePG) and egg-derived phosphatidyl-choline (ePC) were used to mimic the negatively charged cytoplasmic membrane of bacteria. In contrast, ePC and cholesterol were used to emulate the neutral outer leaflet of eukaryotic membranes [32]. In general, as expected, all Tritrp-derived peptides induced higher levels of calcein leakage in negatively charged LUVs in comparison to in neutral LUVs (Figure 5). Additionally, the Tritrp peptide with Arg residues could induce more calcein leakage than peptides with Lys residues for both LUV systems (Figure 5). 

When peptides with different side chain lengths for Arg and Lys were used, they exhibited different behaviors towards the LUV systems. For the negatively charged LUVs (Figure 5, ePC/ePG), reduction (Tritrp–Agb) and increase (Tritrp–hArg) of the length of the Arg did not significantly affect the calcein leakage. In contrast, an increase in the side chain length of the Lys analogs resulted in a length-dependent increase of the ability to induce calcein leakage in the ePC/ePG LUVs. The leakage percentage increased from ~50% for Tritrp–Dap (one-carbon side chain) to ~80% for Tritrp–Lys (four-carbon side chain). Also, for the neutrally charged LUVs (Figure 5, ePC/Chol), modifications of the Arg side chain length did not result in statistically significant changes in the membrane permeabilization characteristics. Nonetheless, an increase in the side chain length of Lys was now correlated with a decrease in the calcein leakage induced for these LUVs. The presence of the shortest Lys analog, Dap, induced a leakage of 50%, while the presence of the longer Lys residue induced only 20% calcein leakage.

### 2.5. Secondary Structure Determination

**Figure 6:**
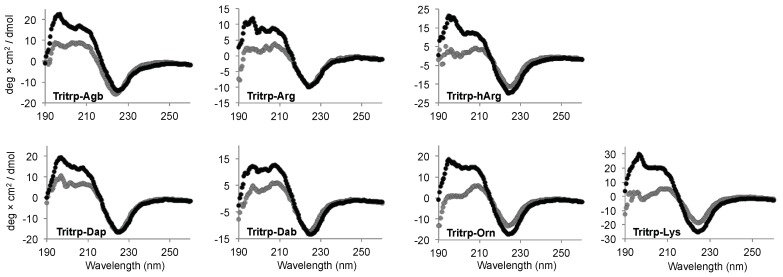
Far-ultraviolet (UV) circular dichroism spectra of several Tritrp-derived peptides in the presence (black) or absence (gray) of SDS micelles. The peptides (50 µM) were incubated in Tris buffer (10 mM) pH 7.5 in the presence or absence of 30 mM SDS.

Interaction of AMPs with lipid membranes is normally coupled to the acquisition of a specific 3D structure. Therefore, we studied the conformational changes induced upon interaction with the SDS micelles for the Tritrp analogs with different side chain lengths by far-ultraviolet (UV) circular dichroism (CD) (Figure 6). The CD spectra for all the peptides in buffer solution were remarkably similar and were characterized by a strong negative signal ~225 nm (Figure 6, gray). Upon binding to the SDS micelles, all peptides retained the strong signal at ~225 nm, while the positive signals between 195–210 nm increased in comparison to the buffer conditions (Figure 6, black). The changes in this region of the spectra suggest a difference in the 3D structure of the peptides due to the binding of the peptide to the SDS micelles.

The CD signal at ~225 nm has been previously allocated to the Trp side chain interactions with the backbone of the peptides as well as to Trp–Trp stacking interactions for AMPs with a high content of Trp residues [33]. In the case of all the Tritrp peptides, the presence of three Trp residues explains the strong signal observed at this wavelength. As a result, a direct evaluation of the secondary structure is not reliable in this case. However, the data showed that all peptides retained a similar secondary structure independent of the side chain length for the Arg and Lys residues for all the Tritrp analogs (Figure 6).

**Figure 7:**
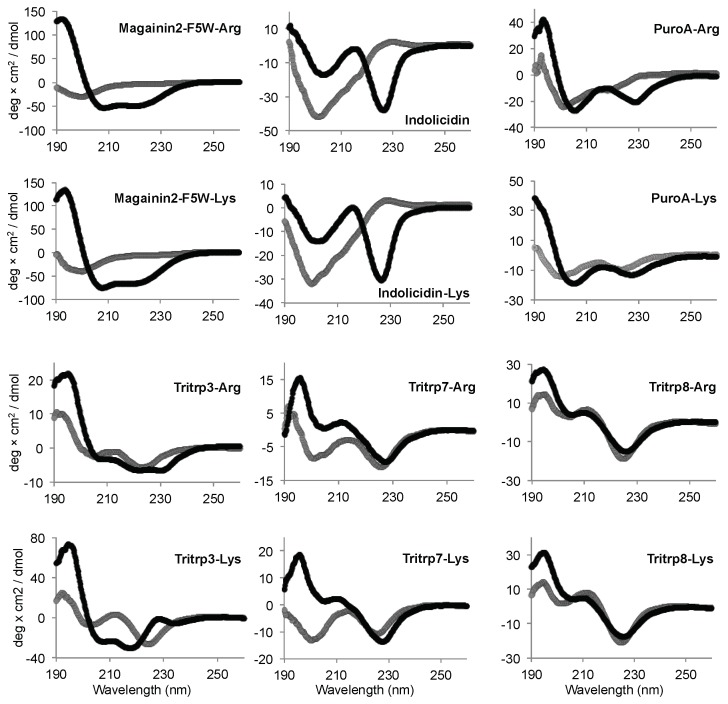
Far-UV circular dichroism spectra of several Trp-rich AMPs in the presence (black) or absence (gray) of SDS micelles. The peptides (50 µM) were incubated in Tris buffer (10 mM) pH 7.5 in the presence or absence of 30 mM SDS. Magainin2-F5W was used as a helical control peptide.

Similar to the Tritrp-derived peptides, the diverse group of Trp-rich AMPs as well as magainin2-F5W also exhibited remarkably comparable CD spectra for the AMP analogs with either Arg or Lys as the sole source of positive charge to the peptide (Figure 7). Interestingly, Tritrp3 exhibited a slightly different CD spectrum when containing either Arg or Lys residues.

### 2.6. Trypsin Degradation

The potential therapeutic use of most AMPs is highly dependent on their stability in complex environments such as human blood, where degradation by proteases can play a major role. An initial assessment of the resistance of the Tritrp analogs to protease degradation was performed in this study by incubating the peptides in the presence of the protease trypsin. The presence of Arg and Lys residues in the Tritrp peptides should render the peptides extremely susceptible to trypsin degradation. The digestion of the peptides could be visualized by reverse-phase chromatography (Figure 7). 

**Figure 8:**
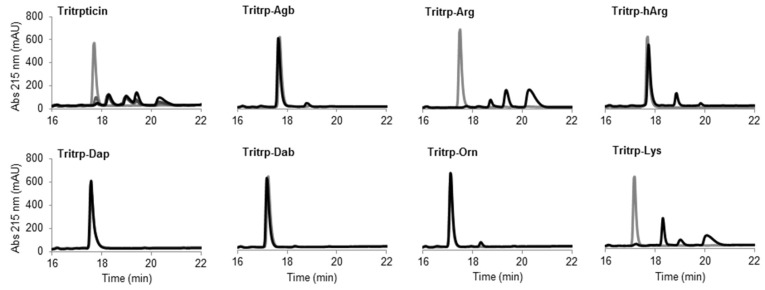
Reverse-phase chromatography of trypsin digestion of AMPs. The peptides (10 µM) were incubated in the presence of trypsin (30 units) at 37 °C. Samples collected at times 0 (gray) and 60 min (black) were analyzed by reversed-phase high-performance liquid chromatography (RP-HPLC). The originally discovered and nonamidated tritrpticin peptide was used as control and analyzed at time intervals of 0, 15, 30, and 60 min (gray to black color scale, respectively). Abbreviation: Abs, absorbance.

The peptide tritrpticin (without C-term amidation) was rapidly hydrolyzed by trypsin. Only 15 min after incubation, most of the peptide was degraded and shorter fragments with higher retention times appeared (Figure 8 Tritrpticin, dark gray). After a one-hour incubation, the degradation pattern of tritrpticin under these conditions seemed complete (Figure 8 Tritrpticin, black). Therefore, all other peptides were digested for one hour before reversed-phase high-performance liquid chromatography (RP-HPLC) analysis. The amidated Tritrp-peptides with Arg and Lys were completely hydrolyzed by trypsin as indicated by the disappearance of the main peptide peak (gray) and the generation of smaller fragments (black). The reduction of the length of the side chain of Arg by only one carbon (Tritrp–Agb) resulted in a remarkable increase in the resistance to trypsin degradation. After a one-hour incubation, only a small peak was detected at a higher retention times. Similarly, elongation of the Arg side chain resulted in a peptide analog (Tritrp–hArg) with improved stability but the effect was slightly lower than for Tritrp–Agb.

In the case of the different side chain lengths for Lys, considerable changes were also observed. Sequential reduction of the side chain length resulted in peptides with high resistance to trypsin hydrolysis. Removal of one carbon (Tritrp–Orn) was already associated with a considerable increase in stability, similar to Tritrp–Agb. Further removal of carbons was followed by an even higher stability (Tritrp–Dab). Finally, the peptide Tritrp–Dap resulted in no detectable degradation of the peptide after one hour of incubation.

## 3. Discussion

Peptide–membrane interactions have been identified as a key element for the antimicrobial action of most AMPs [2]. During the initial complex formation between AMPs and biological/synthetic membranes, electrostatic interactions between negatively charged membranes and the cationic peptides play a crucial role [10]. Thus, the amino acid residues responsible for the positive charge of AMPs are of particular interest. In this work, the differences in the biological activity and the membrane permeabilization properties of several Trp-rich AMPs containing either Arg or Lys residues were evaluated. Additionally, the effect of the side chain length of these residues on the activity of the peptides and their resistance to tryptic degradation were also studied. To the best of our knowledge, a comprehensive study analyzing different AMPs and correlating their antimicrobial activity, structure, mechanism, and protease susceptibility has not yet been reported.

In the current study, three different Trp-rich AMPs were selected (tritrpticin, indolicidin, and puroA), while magainin2-F5W was used as a control. These peptides have been extensively studied in terms of their mechanism of action and structural characteristics [27,30,31,34,35,36,37,38]. The four peptides exhibit different amino acid sequences as well as distinctive structures when interacting with SDS or dodecylphosphocholine (DPC) micelles (Table 1). In order to identify the changes induced by the chemical nature of the positively charged residues on the AMPs, these residues were systematically substituted to obtain peptides containing either Arg or Lys (Table 2). 

The antibacterial activity of almost all peptides (Tritrp, magainin2-F5W, and indolicidin) was diminished by the presence of Lys residues. However, the changes were relatively small for magainin2-F5W and indolicidin when compared to Tritrp–Lys. This last peptide experienced a fourfold decrease in antibacterial activity in comparison to Tritrp–Arg. These results are in accordance with reported antibacterial activities of tritrpticin and SYM11 (a symmetrical derivate of tritrpticin) [39], and other peptides such as temporin-1Tl, bactenecins, and cyclic CC_2_β_2_ [21,40,41,42]. Taken together, these data indicate that Lys residues can negatively influence the bacterial killing properties of AMPs. In terms of their mechanism of action, all peptides could induce cytoplasmic membrane permeabilization in *E. coli* at their MIC or higher (Figure 2, Figure 3 and Figure 4). The level of permeabilization induced at a fixed concentration (e.g., 8 µM) correlated with the antibacterial activity of the peptides. Indeed, for most Arg-containing peptides, the level of cytoplasmic membrane permeabilization was consistently higher than for the Lys-containing peptides. Additionally, the permeabilization of artificial membranes, such as LUVs, also indicated that peptides containing guanidinium side chain groups were more effective at disrupting phospholipid bilayers in contrast to peptides containing Lys-derived side chains. Other studies have reported similar behaviors [24,39], and suggested that the larger hydrogen bonding capabilities of the guanidinium with the phosphodiester groups of phospholipids, together with their ability to rigidly organize and cluster these groups, is responsible for the stronger membrane disruption properties of Arg-containing peptides in comparison to Lys-containing peptides [22,23,43].

The secondary structure of the peptides bound to biological membranes could also potentially influence the biological activity of the peptides when Arg residues are substituted by Lys. However, the CD spectra for most peptides did not depend on the presence of Arg or Lys residues, because similar patterns of ellipticity were observed upon binding to SDS micelles (Figure 6 and Figure 7). This suggests that upon interaction with micelles the Arg- or Lys-containing peptides acquire a similar secondary structure. Interestingly, Tritrp3 was the only peptide for which substantial differences in the CD spectra were observed between the Arg and Lys versions. However, these two peptides (Tritrp3–Arg and Tritrp3–Lys) did not exhibit any difference in their antimicrobial potency. Consequently, secondary structural changes, as evaluated by CD spectroscopy, could not explain the observed differences in the antibacterial activity or the ability to induce permeabilization in synthetic and *E. coli* cytoplasmic membranes. It is important to note that for most Trp-rich peptides, the strong CD signal between 210 and 240 nm, which is correlated with the high content of Trp residues, could mask certain structural differences among the SDS-bound peptides. This Trp interference on the secondary structure determination of Trp-rich peptides by CD spectroscopy has been previously reported [33]. In addition to CD spectroscopy, other spectroscopy techniques such as Fourier-transform infrared spectroscopy (FTIR) or solution nuclear magnetic resonance (NMR) spectroscopy can be used to obtain structural information for micelle-bound peptides [25,33]. In fact, the 3D structures for the Tritrp–Arg and Tritrp–Lys peptides have already been solved in our laboratory by ^1^H-NMR spectroscopy, and very similar DPC-bound structures were determined for these two peptides [25]. In combination with our current CD spectroscopy results, the previously reported NMR data suggest that structural differences between these two Tritrp peptides (Arg or Lys) are unlikely to be responsible for the observed differences in biological activity. Similar structural NMR studies could be performed in the future for the other peptide analogs with altered Arg/Lys side chain length. 

Additionally, and due in part to the aforementioned limitations of the use of CD spectroscopy, we also considered the originally reported NMR structures for all the other AMPs used in this study when interacting with SDS or DPC micelles (Table 1). Again, there was no clear correlation between the structures and biological activities of magainin2-F5W (α-helix), indolicidin (β-turns), puroA (helical/β-turn), and Tritrp1 (helical/β-turn) when Arg or Lys peptides were compared. Due to the considerable differences in amino acid sequence among these AMPs, an additional set of three peptides derived from Tritrp was investigated to further study the relationship between secondary structure and antimicrobial activity. Tritrp3, Tritrp7, and Tritrp8 are analog peptides with different secondary structures when bound to DPC micelles [25]. Tritrp3 is characterized by a fully α-helical structure, which occurs due to the substitution of two Pro by Ala residues in Tritrp. In the peptides Tritrp7 and Tritrp8, only one Pro residue is substituted by Ala, resulting in a reduced helical content (Table 1). The antibacterial activity of Tritrp3 (α-helix) was not affected (MIC 2 µM) by the use of either Arg or Lys in its sequence. This observation is in marked contrast to the activity of the original Tritrp1 peptide (β-turns). Interestingly, the reduction of the α-helical content in the Tritrp7 was correlated with changes in the activity of the peptides to the presence of Arg or Lys residues (Table 2). However, reduction of the α-helical content on the Tritrp8 peptide did not exhibit the same behavior. Therefore, a direct relationship between the peptide’s secondary structure and the biological activities of Arg- or Lys-containing AMPs was not evident.

Since the Tritrp–Arg/–Lys peptides exhibited the largest difference in biological activity, the side chain lengths of the Arg and Lys residues in these peptides were systematically changed and the effects of these modifications were analyzed. The antibacterial activity of the peptides was essentially unaltered by a reduction (Agb) or increase (hArg) in the side chain length of Arg analogs (Table 2). These results are in agreement with the unchanged antibacterial activity reported for guanidinium-functionalized polycarbonate polymers with different spacer lengths for the guanidinium groups ranging from 2 to 5 carbon atoms [44]. In addition, these results correlated well with the liposome permeabilization effects induced by the peptides (Figure 3). In contrast, sequential reduction of the number of hydrocarbons in the side chain of Lys residues was linked to a fourfold increase of the antibacterial activity (Table 2). However, there was no correlation between the membrane permeabilization of the negatively charged ePC/ePG LUVs and the antimicrobial activity, because the calcein leakage was decreased by the presence of shorter versions of Lys (Figure 5). Remarkably, the membrane permeabilization of neutrally charged membranes (ePC/Chol) did correlate with the antimicrobial activity of peptides containing Lys residues of varying length (Figure 5). Clearly, the membrane composition plays a very important role in the activity of the peptides carrying different Lys analogs.

Most of the Arg- and Lys-derivative peptides exhibited a strong correlation between the severity of cytoplasmic membrane disruption in *E. coli* and antibacterial activity. However, when the Lys residue was shortened by three hydrocarbons, the peptide Tritrp–Dap exhibited the strongest antibacterial activity but the poorest membrane-lytic ability. Taken together, the results from the artificial and *E. coli* membrane studies suggest that the mechanism of action involving membrane permeabilization of the peptides can be modulated and, in certain instances, could be altered by changes in the side chain length of positively charged residues. Interestingly, a similar increase in antimicrobial activity due to substitution of Lys by Dap was not found for the antimicrobial peptide C18G (containing 7 Lys residues) [45]. Nonetheless, both peptides (Tritrp–Dap and C18G–Dap) exhibited a reduction in their ability to permeabilize the inner membrane of *E. coli* [45]. On the other hand, studies of amphiphilic random copolymers with different cationic side-chain spacer arms (2-aminoethylene, 4-aminobutylene, or 6-aminohexylene) also established that the reduction of the side chain length had a profound effect on their antimicrobial activity [46]. 

In order to evaluate susceptibility to proteolytic degradation, the stability of the Tritrp analogs upon incubation with trypsin was evaluated. As expected, trypsin could rapidly (<60 min) degrade the Tritrp–Arg and Tritrp–Lys peptides (Figure 8). Remarkably, any modification in the length of the Arg or Lys residues had a positive effect on the resistance of the peptides against trypsin hydrolysis. In the case of the Arg analogs, the protection was not complete, because a small amount of peptide fragments were still detected after trypsin incubation. Nonetheless, for the Lys analogs, a higher degree of protection was provided with the sequential reduction of the side chain length. The shorter Lys analogs do not fit in the trypsin specificity pocket, thereby preventing peptide hydrolysis. In addition to the change in the accessibility of the Arg and Lys residues to the active site of trypsin, it has also been reported that the overall structure of the peptides can also be affected by the Arg and Lys analogs, thus altering the interaction between the peptides and the protease [15]. However, the CD spectra of the Tritrp analogs with different Arg/Lys side chain lengths in aqueous environment did not provide an explanation for the differences in the proteolysis stability of these peptides (Figure 8). All peptides exhibited similar CD spectra when incubated in buffer solution, likely representing a combination of several conformers as detected by NMR spectroscopy for the Tritrp peptide [25]. Schibli et al. [25] have shown by 1D ^1^H-NMR that in aqueous solution the peptides Tritrp–Arg and Tritrp–Lys exhibited at least three distinct conformations, as determined by differences in the Hε1-Trp resonances. Hence, the CD signals reported in this study should be considered as the average ellipticity of different peptide conformers. This peptide flexibility, explained in part by cis–trans isomerization of the Pro residues could support the proposed link between the conformational flexibility and protease degradation resistance described by Bagheri et al. [21] for bactenecin-derived peptides. 

In conclusion, in most cases, the presence of Arg (guanidinium side chain group) or Lys (amino side chain group) as the exclusive source of positive charges had a considerable impact on the antibacterial activity of the AMPs studied here. Most peptides containing Arg and Arg derivatives exhibited higher antimicrobial activities than did the Lys- and Lys-derivative-containing peptides. The antibacterial activity correlated with the ability of the peptides to disturb the inner membrane of *E. coli* and the synthetic LUV phospholipid bilayers, but was not dependent on side chain length. On the other hand, changes to the length of the side chain increased the antibacterial activity of the Lys-containing peptides. However, the mechanism of action for the peptides with shorter side chains only correlated with high ability to permeabilize the membrane of *E. coli*. Lastly, the peptide’s susceptibility to hydrolysis by trypsin was drastically improved by the reduction of the side chain length for both the Arg- and Lys-containing peptides. Altogether, our results illustrate the different effects derived from the chemical nature of the positively charged residues, and how these residues can be used to modulate the activity of diverse AMPs. Most importantly, reduction of the side chain length of Lys residues also appears to provide a potential new mechanism for improving the antibacterial activity in parallel with increasing the stability of the peptides towards trypsin degradation. 

## 4. Materials and Methods

### 4.1. Materials, Peptides, and Bacterial Strains

Peptides were chemically synthesized by standard solid-phase methodology, and purity (>95%) and molecular weight were confirmed by reverse-phase chromatography and mass spectrometry, respectively. Tritrp–Dap was provided by PolyPeptide Group (San Diego, CA, USA) and all remaining peptides were manufactured by GenScript, Inc. (Piscataway, NJ, USA). Melittin was purified from honey bee venom (>70% purity) and was provided by Sigma Aldrich (St. Louis, MO, USA). The peptide concentrations were determined by absorbance at 280 nm, using the extinction coefficient provided by the ProtParam tool from the ExPASy server [47]. 

Cholesterol (Chol), l-α-phosphatidylcholine (ePC), and l-α-phosphatidylglycerol (ePG) from chicken eggs were provided as chloroform stocks by Avanti Polar Lipids, Inc. (Alabaster, AL, USA). Luria-Broth was purchased from BioShop Canada Inc. (Burlington, Canada). Trypsin from bovine pancreas and all other chemical reagents were purchased from Sigma-Aldrich (St. Louis, MO, USA).

*Escherichia coli* ATCC 25922 was purchased from the American Type Culture Collection (Manassas, VA, USA) and *E. coli* ML35p was kindly provided by Dr. Robert Lehrer from the David Geffen School of Medicine at UCLA (Los Angeles, CA, USA).

### 4.2. Antibacterial Activity

The antibacterial activity for the peptides in this study against *E. coli* ATCC 25922 was determined by the broth microdilution method [48]. Briefly, *E. coli* cells at 5 × 10^5^ cfu/mL, obtained from a single colony culture, were incubated in Mueller Hinton broth (MHB) media (Sigma Aldrich, St. Louis, MO, USA) in the presence of two-fold dilution of peptides (0–256 µM) in 96-well polypropylene microtiter plates (Sigma Aldrich, St. Louis, MO, USA). The plates were incubated at 37 °C for 16 h, and the minimal inhibitory concentration was determined after incubation as the minimal peptide concentration at which cell growth was not observed. 

### 4.3. Escherichia coli ML35p Inner Membrane Permeabilization

Permeabilization of the inner membrane of *E. coli* by AMPs and their analogs was measured as described by Epand et al. [49]. This method requires the *E. coli* strain ML35p, which, in addition to constitutively expressing the cytoplasmic enzyme β-galactosidase, lacks the *lac* permease, preventing the entrance of β-galactosides. In the case of outer and inner (cytoplasmic) membrane permeabilization (e.g., induced by AMPs), impermeable β-galactosidase substrates, such as 2-nitrophenyl-β-galactopyranose (ONPG) (Sigma Aldrich, St. Louis, MO, USA), can reach the cytoplasm and are hydrolyzed by the enzyme. This hydrolysis can be followed by absorbance at the 420 nm wavelength.

*Escherichia coli* ML35p was grown in Luria-broth (LB) media at 37 °C, from a single colony, until OD_600_ ~0.6. The cells were then washed twice and finally resuspended in assay buffer (Na^+^-phosphate 10 mM pH 7.5, NaCl 100 mM, and LB 300 µg/mL). The resuspended cells were incubated at a final OD_600_ = 0.3 in the presence of ONPG (0.5 mM) and two-fold dilutions of AMPs in the assay buffer, in a 96-well microtiter plate at 37 °C. The absorbance at 420 nm was measured every 2 min for 60 min in a plate reader Eppendorf AF2200 (Eppendorf, Mississuaga, Canada) with temperature control (37 °C) and orbital plate shaking (10 seconds before each reading). The highest concentration of peptides corresponded to 4 × MIC (Table 1). 

### 4.4. Large Unilamellar Vesicles and Calcein Leakage

Large unilamellar vesicles (LUVs) composed of ePG and ePC at a 1:1 molar ratio, as well as ePC and cholesterol at a 2.5:1 molar ratio, were prepared by the extrusion method as described previously [32,50]. Briefly, appropriate amounts of lipids and/or cholesterol were mixed in glass vials, and the organic solvent was evaporated under a constant flow of nitrogen. Remaining solvent was removed by vacuum overnight. The dry lipids were resuspended in leakage buffer (Tris 10 mM pH 7.5, NaCl 150 mM, and ethylenediaminetetra-acetic acid (EDTA) 1 mM) and supplemented with calcein 70 mM (Sigma Aldrich, St. Louis, MO, USA) by vortexing; the suspension was then freeze-thawed seven times using liquid nitrogen and warm (~40 °C) water. LUVs of 100 nm in diameter were formed by passing the solution through 0.1 µM polycarbonate filters (Nucleopore filtration products, Pleasonton, CA, USA). Free calcein was removed by size exclusion chromatography using a Sephadex G-50 column (Sigma Aldrich, St. Louis, MO, USA). The concentration of lipids in the LUVs was determined by the Ames phosphate assay [51], performed in triplicate.

LUV membrane permeabilization was measured as leakage from calcein-containing vesicles. Calcein leakage was followed by fluorescence spectroscopy in a Varian Cary Eclipse fluorimeter (Agilent technologies, Santa Clara, CA, USA). LUVs (2.0 µM) were incubated in leakage buffer for 1 min at 25 °C with constant stirring, followed by addition of peptides (0.2 µM), and incubated further for 10 min. At this point, triton X-100 (0.02%) (Sigma Aldrich, St. Louis, MO, USA) was added in order to induce 100% calcein leakage. The kinetics of released calcein fluorescence were measured by excitation and emission wavelengths of 490 and 520 nm, respectively, with bandwidth slits of 5 nm. The percentage of leakage was calculated by Equation (1):

(1) $$\%\;Calcein\;leakage=\;100\;\times \;\frac{\left(I-I_{o}\right)}{\left(I_{t}-I_{o}\right)},$$

where *I* represents the fluorescence after 10 min incubation with the peptide, and *I_t_* and *I_o_* are the fluorescence after 1 min incubation in leakage buffer and in triton X-100, respectively.

### 4.5. Far-UV Circular Dichroism

The secondary structure of the peptides (50 µM) was initially assessed in buffer (Tris 20 mM, pH 7.5) followed by the addition of SDS (35 mM) (Sigma Aldrich, St. Louis, MO, USA) in a Jasco J-810 spectropolarimeter (Jasco Inc, Easton, PA, USA). The far-UV spectra were registered at 190–260 nm in a 1 mm pathlength cuvette at 25 °C. The ellipticity was registered every 0.5 nm, with a scan rate of 100 nm/min and a bandwidth of 0.1 nm. The final spectrum was the average of 10 consecutive scans.

### 4.6. Trypsin Hydrolysis of Antimicrobial Peptides

The resistance of the peptides to degradation by trypsin was assessed following the protocol of Bagheri and Hancock [15], with some modifications. Fresh trypsin solution was prepared by resuspending the lyophilized enzyme (11900 units/mg) in ammonium bicarbonate buffer ((NH_4_)HCO_3_ 100 mM, pH 8.2) to 2.4 × 10^3^ units/mL. Peptides (10 µM) in the same enzyme buffer were incubated with trypsin (30 units). Samples (0.4 mL) were taken at times 0, 15, 30, and 60 min for the peptide tritrpticin, while for all other peptides, only 0 and 60 min samples were collected. The trypsin digestion was stopped by addition of acetic acid/ trifluoroacetic acid (TFA) 2.5% solution (Sigma Aldrich, St. Louis, MO, USA) to 20% (*v*/*v*) at each time point. All samples were then stored at −20 °C until they were analyzed by reverse-phase chromatography using a 5C_18_-AR-300 Cosmosil column (Nacalai Tesque, Inc., Tokyo, Japan) with an acetonitrile/water gradient supplemented with TFA 0.05% (*v*/*v*) in an ÄKTA purifier (GE Healthcare, Pittsburg, PA, USA).

## Figures and Tables

**Table 1:** Sequences and characteristics of the antimicrobial peptides (AMPs) used in this study.

Peptide	Sequence	Net Charge ^a^	3D Structure ^b^	Mechanism of Action	Ref.
Magainin2	GIG**K**FLHSA**KK**FG**K**AFVGEIMNS	+3	α-helix (I2–N22)	Membranolytic	[26,27]
Indolicidin	ILPW**K**WPWWPW**RR**-NH_2_	+4	β-turns (K5, W8)	Intracellular target	[28,29]
PuroA	FPVTW**K**WW**K**WW**K**G-NH_2_	+5	β-turn (T4–W7) helical turn (W8–W10)	Membranolytic	[30,31]
Tritrp1 ^c^	V**RR**FPWWWPFL**RR**-NH_2_	+5	β-turn (P5–W8) helical turn (W9–R12)	Membranolytic	[25]
Tritrp3	V**RR**FAWWWAFL**RR**-NH_2_	+5	α-helix (F4–L11)	Membranolytic	[25]
Tritrp7	V**RR**FAWWWPFL**RR**-NH_2_	+5	α-helix-like (F4–L11)	Membranolytic	[25]
Tritrp8	V**RR**FPWWWAFL**RR**-NH_2_	+5	β-turn (P5–W8) helical turn (W9–R12)	Membranolytic	[25]

^a^ Net charge is calculated for physiological conditions; ^b^ 3D structure determined by nuclear magnetic resonance (NMR) spectroscopy in the presence of sodium dodecyl sulfate (SDS) or dodecylphosphocholine (DPC) micelles; ^c^ C-terminal amidated version of the peptide tritrpticin.

**Table 2:** Peptide sequence and minimal inhibitory concentration (MIC) against *Escherichia coli* ATCC 25922 in Mueller-Hinton broth media. The MICs were run in triplicate and the range of all three experiments is presented.

Peptide	Sequence	MIC (µM)
Magainin2-F5W–Arg	GIGRFLHSARRFGRAFVGEIMNS	8
Magainin2-F5W–Lys	GIGKFLHSAKKFGKAFVGEIMNS	16
Indolicidin	ILPWKWPWWPWRR-NH_2_	8
Indolicidin–Lys	ILPWKWPWWPWKK-NH_2_	16
PuroA–Arg	FPVTWRWWRWWRG-NH_2_	8–16
PuroA–Lys	FPVTWKWWKWWKG-NH_2_	8
Tritrp3–Arg	V R R FAWWWAFL R R -NH_2_	2
Tritrp3–Lys	V K K FAWWWAFL K K -NH_2_	2
Tritrp7–Arg	V R R FAWWWPFL R R -NH_2_	8
Tritrp7–Lys	V K K FAWWWPFL K K -NH_2_	32
Tritrp8–Arg	V R R FPWWWAFL R R -NH_2_	2–4
Tritrp8–Lys	V K K FPWWWAFL K K -NH_2_	4
Tritrp–Agb	V (Agb) (Agb) FPWWWPFL (Agb) (Agb) -NH_2_	4
Tritrp–Arg	V R R FPWWWPFL R R -NH_2_	2–4
Tritrp–hArg	V (hArg)(hArg)FPWWWPFL(hArg)(hArg) -NH_2_	4
Tritrp–Dap	V (Dap) (Dap) FPWWWPFL (Dap) (Dap) -NH_2_	4
Tritrp–Dab	V (Dab) (Dab) FPWWWPFL (Dab) (Dab) -NH_2_	4–8
Tritrp–Orn	V (Orn) (Orn) FPWWWPFL (Orn) (Orn) -NH_2_	16
Tritrp–Lys	V K K FPWWWPFL K K -NH_2_	16
Melittin	GIGAVLKVLTTGLPALISWIKRKRQQ-NH_2_	2
